# Supplementary material for: Bayesian refinement of protein structures and ensembles against SAXS data using molecular dynamics
Source: PLoS Comput Biol. 2017 Oct 18;13(10):e1005800. doi: 10.1371/journal.pcbi.1005800 (PMC5662244; doi:10.1371/journal.pcbi.1005800)
Supplement: S1 Archive — (BZ2) [file pcbi.1005800.s011.tar.bz2 › gromacs_4.62_bayesian_saxs_refi/share/html/online/xpm.html]

xpm file format


## xpm file format

|  |  |
| --- | --- |
| Main Table of Contents | **VERSION 4.6** |
| GROMACS homepage | **Sat 19 Jan 2013** |

---

### Description

The GROMACS xpm file format is compatible with the XPixMap format
and is used for storing matrix data.
Thus GROMACS xpm files can be viewed directly with programs like XV.
Alternatively, they can be imported into GIMP and scaled to 300 DPI,
using strong antialiasing for font and graphics.
The first matrix data line in an xpm file corresponds to the last matrix
row.
In addition to the XPixMap format, GROMACS xpm files may contain
extra fields. The information in these fields is used when converting
an xpm file to EPS with
xpm2ps.
The optional extra field are:

- Before the gv\_xpm declaration: title, legend,
  x-label, y-label and type,
  all followed by a string.
  The legend field determines the legend title.
  The type field must be followed by "continuous" or
  "discrete",
  this determines which type of legend will be drawn in an EPS file,
  the default type is continuous.- The xpm colormap entries may be followed by a string, which is a label for
    that color.- Between the colormap and the matrix data, the fields
      x-axis and/or y-axis
      may be present followed by the tick-marks for that axis.

The example GROMACS xpm file below contains all the extra fields.
The C-comment delimiters and the colon in the extra fields are optional.

```
/* XPM */
/* This matrix is generated by g_rms. */
/* title:   "Backbone RMSD matrix" */
/* legend:  "RMSD (nm)" */
/* x-label: "Time (ps)" */
/* y-label: "Time (ps)" */
/* type:    "Continuous" */
static char * gv_xpm[] = {
"13 13   6 1",
"A  c #FFFFFF " /* "0" */,
"B  c #CCCCCC " /* "0.0399" */,
"C  c #999999 " /* "0.0798" */,
"D  c #666666 " /* "0.12" */,
"E  c #333333 " /* "0.16" */,
"F  c #000000 " /* "0.2" */,
/* x-axis:  0 40 80 120 160 200 240 280 320 360 400 440 480 */
/* y-axis:  0 40 80 120 160 200 240 280 320 360 400 440 480 */
"FEDDDDCCCCCBA",
"FEDDDCCCCBBAB",
"FEDDDCCCCBABC",
"FDDDDCCCCABBC",
"EDDCCCCBACCCC",
"EDCCCCBABCCCC",
"EDCCCBABCCCCC",
"EDCCBABCCCCCD",
"EDCCABCCCDDDD",
"ECCACCCCCDDDD",
"ECACCCCCDDDDD",
"DACCDDDDDDEEE",
"ADEEEEEEEFFFF"
```

---

http://www.gromacs.org
